# The histone deacetylase inhibitor, romidepsin, as a potential treatment for pulmonary fibrosis

**DOI:** 10.18632/oncotarget.17114

**Published:** 2017-04-14

**Authors:** Franco Conforti, Elizabeth R. Davies, Claire J. Calderwood, Thomas H. Thatcher, Mark G. Jones, David E. Smart, Sumeet Mahajan, Aiman Alzetani, Tom Havelock, Toby M. Maher, Philip L. Molyneaux, Andrew J. Thorley, Teresa D. Tetley, Jane A. Warner, Graham Packham, A. Ganesan, Paul J. Skipp, Benjamin J. Marshall, Luca Richeldi, Patricia J. Sime, Katherine M.A. O'Reilly, Donna E. Davies

**Affiliations:** ^1^ The Brooke Laboratory, Clinical and Experimental Sciences, University of Southampton Faculty of Medicine, University Hospital Southampton, Southampton, UK; ^2^ NIHR Respiratory Biomedical Research Unit, University Hospital Southampton, Southampton, UK; ^3^ Department of Medicine/Pulmonary & Critical Care, University of Rochester, Rochester, NY, USA; ^4^ Institute for Life Sciences, University of Southampton, Highfield, UK; ^5^ University Hospital Southampton, Southampton, UK; ^6^ NIHR Respiratory Biomedical Research Unit, Royal Brompton Hospital, London, UK; ^7^ National Heart & Lung Institute, Faculty of Medicine, Imperial College, London, UK; ^8^ Cancer Sciences, University of Southampton Faculty of Medicine, University Hospital Southampton, Southampton, UK; ^9^ School of Pharmacy, University of East Anglia, Norwich, UK; ^10^ Respiratory Medicine, Mater Misericordiae University Hospital, Dublin, Ireland; ^11^ School of Medicine and Medical Science, University College, Dublin, Ireland

**Keywords:** pulmonary fibrosis, histone deacetylase Inhibitors, biomarkers, myofibroblasts, epigenomics

## Abstract

Idiopathic pulmonary fibrosis (IPF) is a progressive disease that usually affects elderly people. It has a poor prognosis and there are limited therapies. Since epigenetic alterations are associated with IPF, histone deacetylase (HDAC) inhibitors offer a novel therapeutic strategy to address the unmet medical need. This study investigated the potential of romidepsin, an FDA-approved HDAC inhibitor, as an anti-fibrotic treatment and evaluated biomarkers of target engagement that may have utility in future clinical trials. The anti-fibrotic effects of romidepsin were evaluated both *in vitro* and *in vivo* together with any harmful effect on alveolar type II cells (ATII). Bronchoalveolar lavage fluid (BALF) from IPF or control donors was analyzed for the presence of lysyl oxidase (LOX). In parallel with an increase in histone acetylation, romidepsin potently inhibited fibroblast proliferation, myofibroblast differentiation and LOX expression. ATII cell numbers and their lamellar bodies were unaffected. *In vivo*, romidepsin inhibited bleomycin-induced pulmonary fibrosis in association with suppression of LOX expression. LOX was significantly elevated in BALF of IPF patients compared to controls. These data show the anti-fibrotic effects of romidepsin, supporting its potential use as novel treatment for IPF with LOX as a companion biomarker for evaluation of early on-target effects.

## INTRODUCTION

Idiopathic pulmonary fibrosis (IPF) is a progressive disease, where the median survival has been reported as 2-5 years from diagnosis. This disease is more common in middle-aged adults and its incidence increases with age suggesting that IPF may be a consequence of accelerated aging of the lung [1, 2]. In addition to lung transplantation, two new drugs, pirfenidone (Esbriet^®^) and nintedanib (Ofev^®^) have recently been approved for IPF treatment. Pirfenidone reduces the risk of disease progression [3, 4], while nintedanib reduces the annual decline in forced vital capacity by approximately 50% [5], but both are associated with undesirable side effects [4, 5]. To date, no therapy has successfully halted, much less reversed, the decline in lung function that is a hallmark of IPF. Therefore, additional drugs that may be used as sole agents or in combination with existing therapies are urgently required to improve patient outcomes.

IPF is characterized by multi-focal destruction of the lung architecture, with accumulation of highly active fibroblasts and myofibroblasts that produce excessive amounts of extracellular matrix (ECM) [6] resulting in impaired gas exchange. Studies in a range of fibrotic diseases including IPF have shown that fibroblasts from the affected organ display aberrations in nearly every aspect of the fibroproliferative/fibrogenic response [7, 8]. Reasons for such widespread functional alterations have not been established in detail. However, while the cause(s) of IPF are unknown, the disease has a series of biological abnormalities (genetic and epigenetic alterations) and risk factors (aging, smoking and environmental exposures) in common with cancer [9]. Specifically, epigenetic mechanisms have been proposed to account for the aggressive phenotype of fibrotic fibroblasts [10–12]. Most recently altered expression of histone deacetylases (HDACs) has been reported in IPF lung tissue, especially in fibroblastic foci, the regions of active fibroproliferation [12]. Thus epigenetic alterations, presumably occurring in response to environmental exposures and/or aging, may be the missing link that predisposes the pulmonary fibroblasts for gene expression changes associated with the development of IPF.

Because of HDAC dysregulation in cancer, inhibitors of these enzymes have been developed, and shown to possess potent anti-proliferative activities towards cancer cells [13]. Several studies have demonstrated anti-fibrotic properties for HDAC inhibitors such as trichostatin A (TSA), vorinostat (SAHA) and panobinostat (LBH589) [12, 14–16] which inhibit multiple classes of HDACs. However, we have shown that selective inhibition of class I HDACs using spiruchostatin A (SpA) was sufficient to reduce the proliferation of IPF fibroblasts and suppressed expression of interstitial collagen *in vitro* [17]. Romidepsin is also a class I selective HDAC inhibitor [18, 19], but it is already FDA-approved for treatment of cutaneous T-cell lymphoma (CTCL) with no reported pulmonary toxicity [20, 21]. Romidepsin has also been used in phase II trials in lung cancer [22, 23], demonstrating selectivity towards tumor cells, crucially, appearing relatively harmless to normal lung epithelial cells [22]. Taken together, these findings strongly suggest that romidepsin merits further investigation as a potential therapy for the treatment of fibrosis.

Herein, we investigated the antifibrotic effects of romidepsin *in vitro* and *in vivo*. Since evaluation of novel IPF therapies poses considerable challenges in selecting outcome measures that allow assessment of clinically meaningful effects, we also investigated whether the extracellular enzyme, lysyl oxidase (LOX) has potential to act as a biomarker for evaluation of the anti-fibrotic effects of romidepsin. LOX post-translationally modifies collagens and elastin to enable covalent cross-linking and is induced by the profibrotic mediator, transforming growth factor beta (TGF-β) [24, 25]. A strong association between organ fibrosis and increased LOX activity has been observed [26–29]. We therefore explored the effect of romidepsin on LOX expression *in vitro*, *in vivo* and in IPF bronchoalveolar fluid (BALF). This work has previously been presented in abstract form [30–34].

## RESULTS

### Romidepsin induces histone H3 acetylation

**Figure 1:**
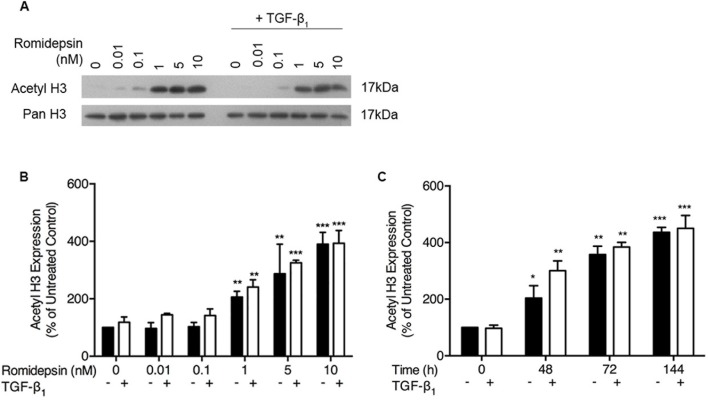
Romidepsin dose and time dependently increased acetylation of histone H3: Fibroblasts were cultured in DMEM/FBS ± TGF-β1 (5ng/ml) with the indicated concentration of romidepsin for 48-144 hours Cell lysates were analysed by SDS-PAGE and western blotting for acetyl- and pan-histone H3. **A**. Representative western blot with **B**. semi-quantitative analysis of the dose-response of IPF fibroblasts to romidepsin at 48h in the absence (solid bars) and presence (open bars) of TGF-β1. **C**. Time-dependent response to 1nM romidepsin ± TGF-β1. Data are shown as mean + SD (*n* = 3; two-way ANOVA with Dunnett's multiple comparisons). *P < 0.05, **P < 0.01, ***P < 0.001. Where there are no lines to indicate the statistical comparison, it is between the starred bar and its equivalent baseline non-romidepsin treated control.

As romidepsin is a histone deacetylase inhibitor, we first assessed histone acetylation in fibroblasts isolated by outgrowth from IPF lung tissue explants. As shown in Figure 1A, romidepsin caused a dose dependent increase in histone H3 acetylation; semi-quantitative analysis demonstrated a doubling in H3 acetylation by 144 hours after treatment with 1nM romidepsin and, with 10nM romidepsin acetylation had increased 4-fold compared to control (Figure 1B). Time-course experiments showed that acetylation was evident after 48h and continued to increase in a time dependent manner. Thus, at 10nM romidepsin, acetylation had doubled by 48h and almost doubled again by 72h. Beyond this time, the acetylation plateaued (Figure 1C). TGF-β_1_ appeared to have little effect on the degree of acetylation caused by romidepsin.

### Romidepsin diminishes fibroblast proliferation and induces cell cycle arrest via CDKN1A expression

**Figure 2:**
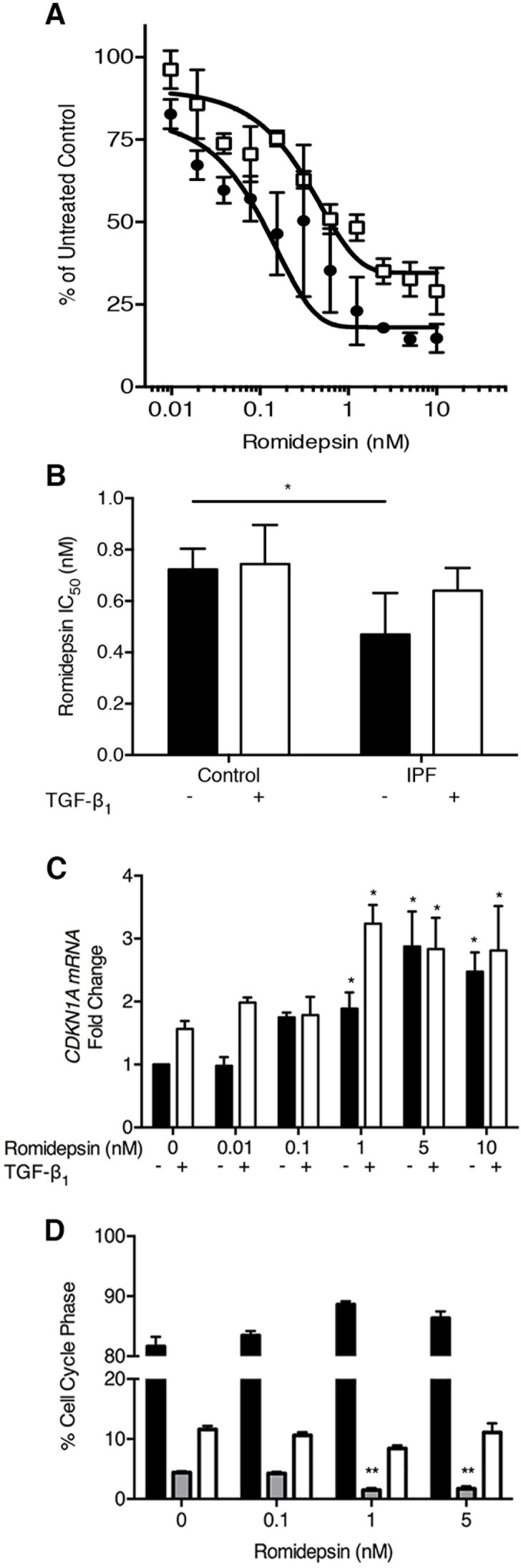
Inhibition of fibroblast proliferation by romidepsin: IPF and normal fibroblasts were cultured for up to 144h in DMEM/FBS ± TGF-β1 (5ng/ml) and ± romidepsin Cells were formalin-fixed before being stained with methylene blue. Stained cells were eluted with a 1:1 ratio of 0.1% HCl and ethanol. Absorbance was measured at 630nm using a photometric plate reader. **A**. Dose response of IPF fibroblasts to romidepsin at 144h in the absence (solid symbols) or presence (open symbols) of TGF-β1. **B**. Comparison of the romidepsin concentration required to achieve 50% growth inhibition (IC50) of the IPF and normal fibroblasts at 144h. **C**. At 48h, after culturing as described, RNA was extracted using the Trizol method prior to cDNA synthesis and analysis by RTqPCR. Data were normalized to the housekeeping genes *UBC/A2* using the ΔΔCT method. *CDKN1A* mRNA expression in response to increasing doses response of romidepsin in IPF fibroblasts without (solid bars) or with (open bars) TGF-β1. Data in **B**. & **C**. are shown as mean + SD (*n* = 3; two-way ANOVA with Dunnett's multiple comparisons). *P < 0.05, **P < 0.01. **D**. Cell cycle analysis of romidepsin treatment of 3 primary IPF fibroblasts cultures quantitatively determined by propidium iodide (PI) flow cytometric analysis. Solid bar = G1, grey bar = S, open bar = G2/M phase. Data are shown as mean + SD (*n* = 3; one-way ANOVA). **P < 0.01. Where there are no lines to indicate the statistical comparison, it is between the starred bar and its equivalent baseline non-romidepsin treated control.

Fibroblasts from IPF lung tissue were cultured in serum-containing medium to optimize their proliferative potential and treated in the absence or presence of TGF-β_1_, which was used to mimic a pro-fibrotic environment. Treatment with romidepsin over 7 days had an inhibitory effect on fibroblast proliferation: the IC_50_ for romidepsin at 144h was 0.5nM in the absence of TGF-β_1_ and 0.6nM in the presence of TGF-β_1_ (Figure 2A). These IC_50_ values for IPF fibroblasts were slightly lower than those of fibroblasts from normal lung parenchyma when treated in an identical manner (Figure 2B).

Several studies, including one in IPF fibroblasts, have suggested that suppression of proliferation by HDAC inhibitors is due to induction of cell cycle arrest involving up-regulation of cell-cycle inhibitors such as *CDKN1A* (p21^waf1^) [17, 35, 36]. We therefore evaluated the effects of romidepsin on *CDKN1A* expression in IPF fibroblasts. At 48h, increased expression was evident at doses of romidepsin as low as 0.1nM. In the absence of TGF-β_1_, a doubling of expression was seen at 1nM romidepsin, while at 5 and 10nM, a 3-fold increase in *CDKN1A* expression was seen. In the presence of TGF-β_1_, induction of *CDKN1A* was observed at baseline and there was a 3-fold increase at 1nM romidepsin (Figure 2C). Romidepsin treatment also induced *CDKN1A* in normal control fibroblasts, but to a lesser extent than those cultured from IPF tissue (163±8% stimulation *vs.* 232±26% at 1nM romidepsin). Cell cycle analysis using FACS confirmed that romidepsin treatment caused fibroblasts to undergo G1 arrest with a significant reduction in the number of cells in S phase at 1nM and 5nM romidepsin (Figure 2D).

### Romidepsin suppresses myofibroblast differentiation

**Figure 3:**
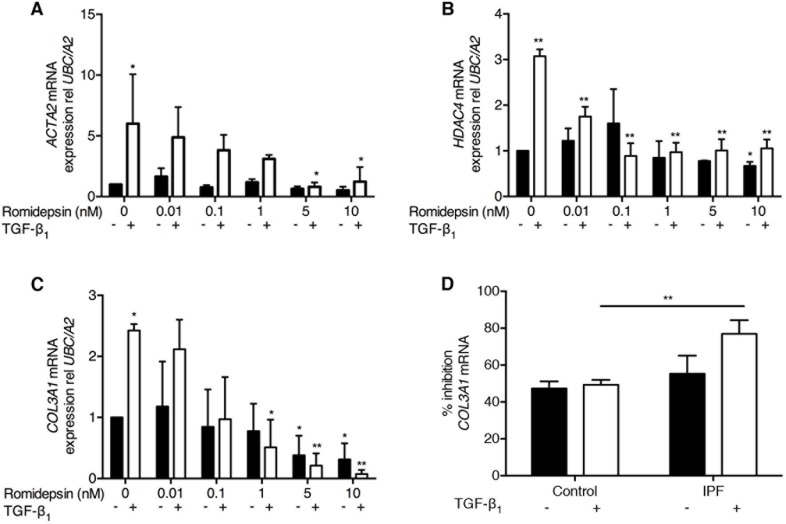
Romidepsin suppressed myofibroblast differentiation: Fibroblasts were cultured in DMEM/FBS ± TGF-β1with the indicated concentration of romidepsin for 48 hours and then samples harvested into Trizol for RNA isolation, cDNA synthesis and RTqPCR analysis Figure shows mRNA expression of **A**. *ACTA2* **B**. *HDAC4* and **C**. *COL3A1* mRNA in IPF fibroblasts in response to romidepsin in the absence (solid bars) or presence (open bars) of TGF-β1. **D**. Inhibition of *COL3A1* mRNA expression by 5nM romidepsin ± TGF-β1 in normal or IPF fibroblasts expressed as a percentage of the corresponding untreated control. Data were normalized to the housekeeping genes *UBC/A2* using the ΔΔCT method. Data are presented as mean + SD (*n* = 3; two-way ANOVA with Dunnett's multiple comparisons). *P < 0.05, **P < 0.01. Where there are no lines to indicate the statistical comparison, it is between the starred bar and its equivalent baseline non-romidepsin treated control.

TGF-β_1_ is a potent stimulus for myofibroblast differentiation that is characterized by induction of α-SMA expression. As expected, TGF-β_1_ promoted expression of α-SMA in IPF fibroblasts with mRNA expression being increased 6-fold by TGF-β_1_. Treatment with romidepsin dose-dependently inhibited this effect, with significant suppression of myofibroblast differentiation at 5nM romidepsin (Figure 3A). Previous studies showed that HDAC4 silencing inhibited myofibroblast differentiation [14]. Consistent with this, we found that TGF-β_1_ up-regulated *HDAC4* mRNA expression in IPF fibroblasts, and this was prevented in the presence of romidepsin (Figure 3B). Inhibition of *HDAC4* expression was evident with doses as low as 0.01nM, while 1nM romidepsin abolished the TGF-β_1_-mediated effect.

A key feature of myofibroblast activity is the ability to synthesize ECM components in response to TGF-β_1_. When IPF fibroblasts were treated with TGF-β_1,_ a 2.5-fold increase in *COL3A1* mRNA expression was observed and romidepsin dose-dependently blocked this effect (Figure 3C). *COL3A1* mRNA expression in control fibroblasts was also inhibited by romidepsin, but the effect was not as marked, particularly in response to TGF-β_1_ where there was significantly less inhibition compared to the fibrotic fibroblasts (Figure 3D).

**Figure 4:**
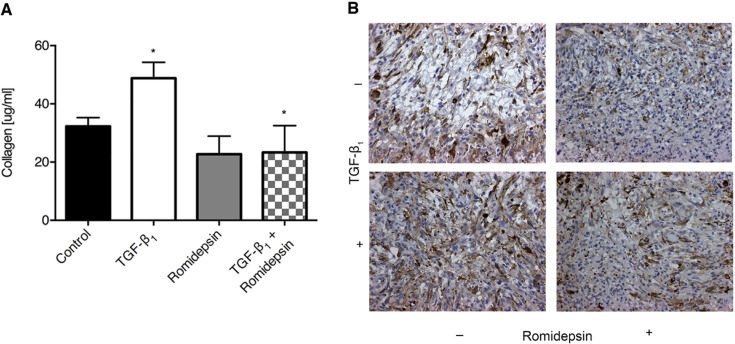
Romidepsin inhibited soluble collagen production and α-SMA expression in 3D pellet culture: 3D pellet cultures were treated without or with 5nM romidepsin in the absence or presence of TGF-β1 for 144h, at which point the pellet was fixed and paraffin embedded **A**. assessment of soluble collagen released by the pellets under the indicated conditions and **B**. representative immunohistochemical staining for α-SMA. In **A**. data are presented as mean + SD (*n* = 3; one-way ANOVA with Dunnett's multiple comparisons). *P < 0.05. Where there are no lines to indicate the statistical comparison, it is between the starred bar and its equivalent baseline non-romidepsin treated control.

To further evaluate the effect of romidepsin, we modeled fibroblastic foci using 3D pellet cultures as described previously [17] and assessed release of soluble collagen and α-SMA expression in response to TGF-β_1_. After 7 days of culture, TGF-β_1_ significantly increased release of soluble collagen into the medium and there was an increase in α-SMA immunostaining in the cells. In the presence of romidepsin, these effects of TGF-β_1_ were reduced (Figure 4A and 4B respectively).

### Romidepsin spares ATII cells

**Figure 5:**
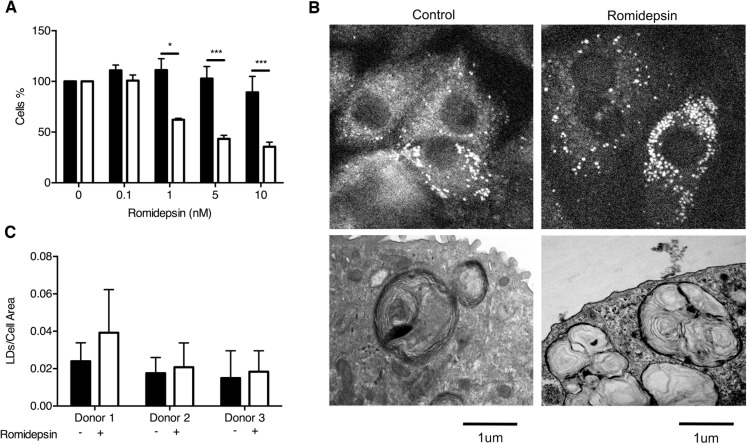
Comparison of the effect of romidepsin on alveolar type 2 cells **A**. The ATII cells (solid bar), isolated from lung resections of 3 different patients, were treated with a range of concentrations of romidepsin, using IPF fibroblasts (open bar) as controls. After 72h, cell proliferation was determined by DAPI staining and direct counting of cell nuclei. **B**. *(Upper panel)* Coherent anti-Stokes Raman spectroscopy of lipids/surfactants in ATII cells alone or after treatment with 5nM romidepsin for 72h. *(Lower panel)* Transmission electron microscopy (TEM) of the ultrastructure of ATII cells cultured on Transwells® for 72h without or with 5nM romidepsin. **C**. CARS image analysis of the pixel ratio of surfactants lipid droplets (LDs) over cell area. In (A & C) data are shown as mean + SD (*n* = 3 donors; two-way ANOVA). * P < 0.05, *** P < 0.001.

ATII cells synthesize and secrete pulmonary surfactant and act as progenitor cells in response to lung injury by trans-differentiating into ATI cells [37]. In view of the potent effects of romidepsin on IPF fibroblasts, we assessed the effect of this drug on primary ATII cells isolated from human lung tissue. Using a direct cell counting assay, ATII cell numbers were unaffected by romidepsin even at doses more than 10x the IC_50_ for fibroblasts; parallel comparison confirmed the effect of romidepsin on IPF fibroblasts in this assay (Figure 5A). To further assess the effects of romidepsin on ATII cell function, we assessed its effects on lamellar body formation. Lipid imaging, by coherent anti-Stokes Raman scattering spectroscopy (CARS) [38], revealed structures comparable in size (1-3μm) to typical ATII cell lamellar bodies. These were present in equal numbers between untreated and treated samples (Figure 5B-5C). Lamellar bodies were also detected by TEM showing osmiophilic organelles characterized by a multi-lamellar structure without any marked difference between treated and untreated samples (Figure 5B). Together these data suggest that romidepsin has minimal effects on ATII cells at doses that markedly suppress fibroblast responses.

### Romidepsin suppresses pulmonary fibrosis in vivo

**Figure 6:**
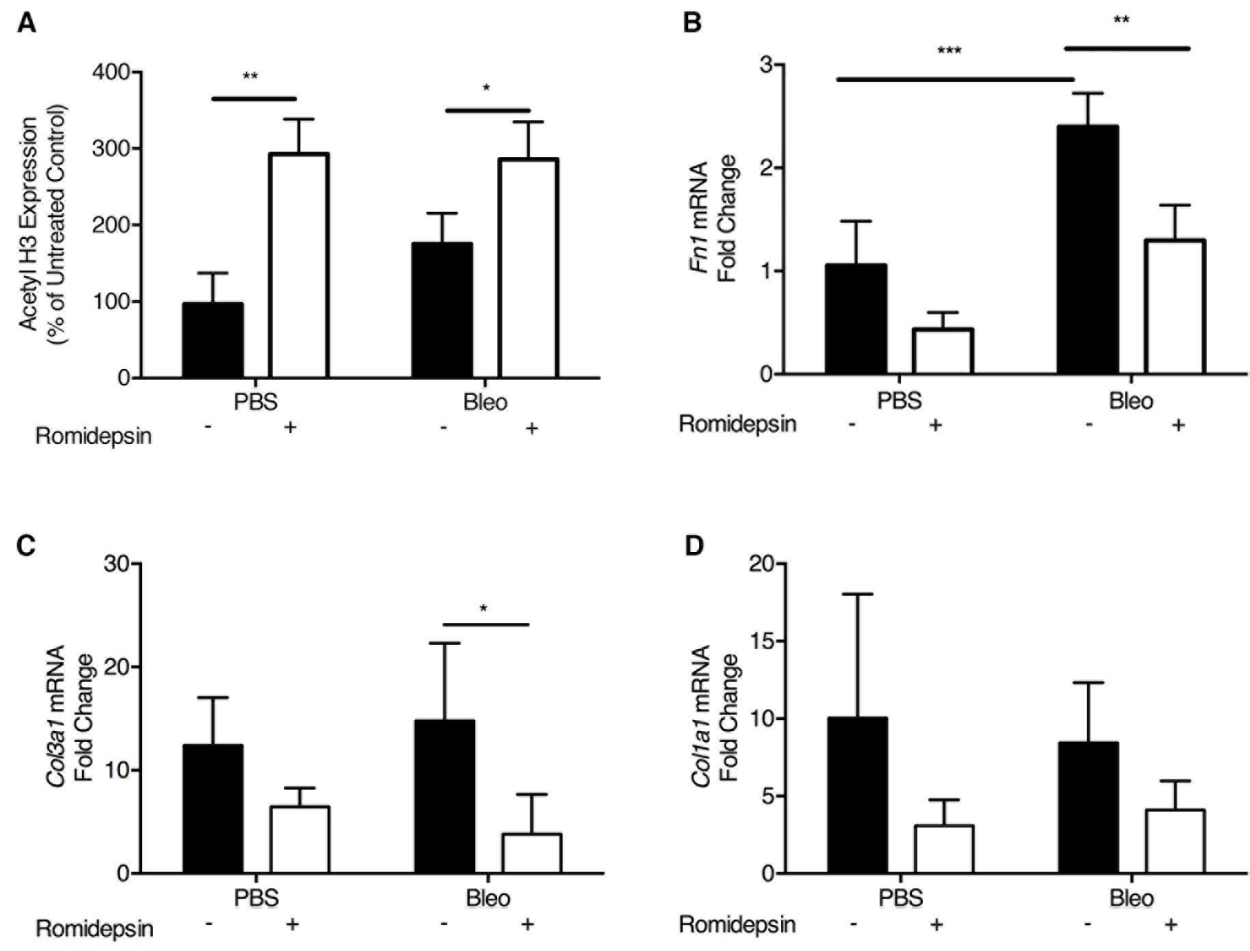
Romidepsin increased histone acetylation and suppressed pro-fibrotic gene expression in bleomycin treated mice: Bleomycin (2U/kg) was instilled *via* the oropharyngeal route to induce lung fibrosis Romidepsin (2mg/kg IP) or a vehicle control was administered 4 days post bleomycin treatment and lungs harvested on day 7 for analysis by western blotting and RTqPCR. **A**. Lungs were homogenized in normal saline with protease inhibitors and lysates analyzed for acetyl histone H3 protein by Western blotting using pan histone H3 as the loading control. mRNA expression for **B**. *Fn1*, **C**. *Col3a1* and **D**. *Col1a1* analyzed by RTqPCR with the gene of interest being normalized to the housekeeping gene *Gapdh* using the ΔΔCT method. Data are presented as mean + SD (*n* = 4 per group within one experiment; two-way ANOVA with Tukey's multiple comparisons). **P < 0.01, ***P < 0.001.

**Figure 7:**
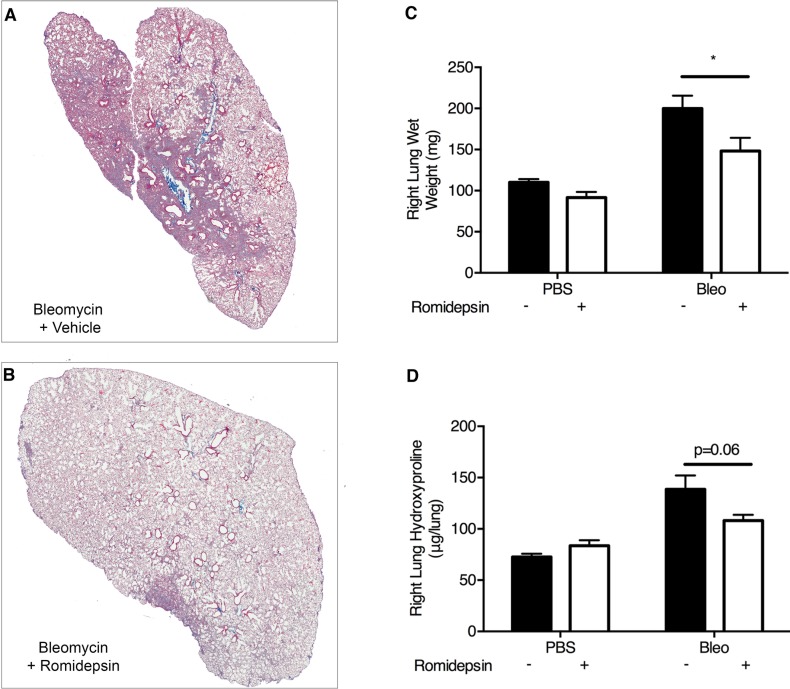
Romidepsin suppressed bleomycin induced pulmonary fibrosis *in vivo*: Bleomycin was instilled *via* the oropharyngeal route to induce lung fibrosis Romidepsin (2mg/kg IP) or vehicle control was administered days 3, 7, 11, and 15 days post bleomycin treatment and the mice were harvested on day 21. **A**. & **B**. Representative images of left lung sections stained with Gomori's trichrome stain to visualize collagen (blue). The right lung was weighed **C**. and then homogenized for determination of hydroxyproline content **D**.. Data are presented as mean + SD (*n* = 6 per group within two experiments; two-way ANOVA with Tukey's multiple comparisons). *P < 0.05.

To test the efficacy of romidepsin as an anti-fibrotic agent *in vivo*, we employed the bleomycin-induced model of pulmonary fibrosis [39, 40]. Initially, we investigated the effect of romidepsin on early changes in gene expression associated with development of pulmonary fibrosis, using groups of mice treated with a single dose of romidepsin (2mg/kg, IP) 3 days after oropharyngeal instillation of bleomycin. Four days later, western blotting of lung lysates revealed that romidepsin treatment had caused a significant increase in H3 acetylation in either PBS or bleomycin-exposed mice (Figure 6A). Romidepsin also significantly prevented up-regulation of pro-fibrotic genes including *Fn1* and *Col3a1* mRNA in the bleomycin-exposed mice, whilst a trend for suppression of *Col1a1* was observed (Figure 6B-6D). Since these short-term experiments confirmed that romidepsin was having the desired effects on histone acetylation and pro-fibrogenic gene expression, we next evaluated its effect on lung pathology using mice that had received bleomycin and were treated with romidepsin or vehicle control on days 3, 7, 11, and 15, with lungs being harvested on day 21. Immunohistochemistry using Gomori's trichrome stain showed that under these conditions, romidepsin reduced fibrosis caused by bleomycin and preserved lung architecture (Figure 7A-7B). Romidepsin also inhibited the bleomycin-induced increase in lung wet-weight (an indicator of increased tissue density and protein accumulation) and collagen content, as assessed by hydroxyproline assay (Figure 7C-7D).

### LOX as a biomarker of romidepsin activity

**Figure 8:**
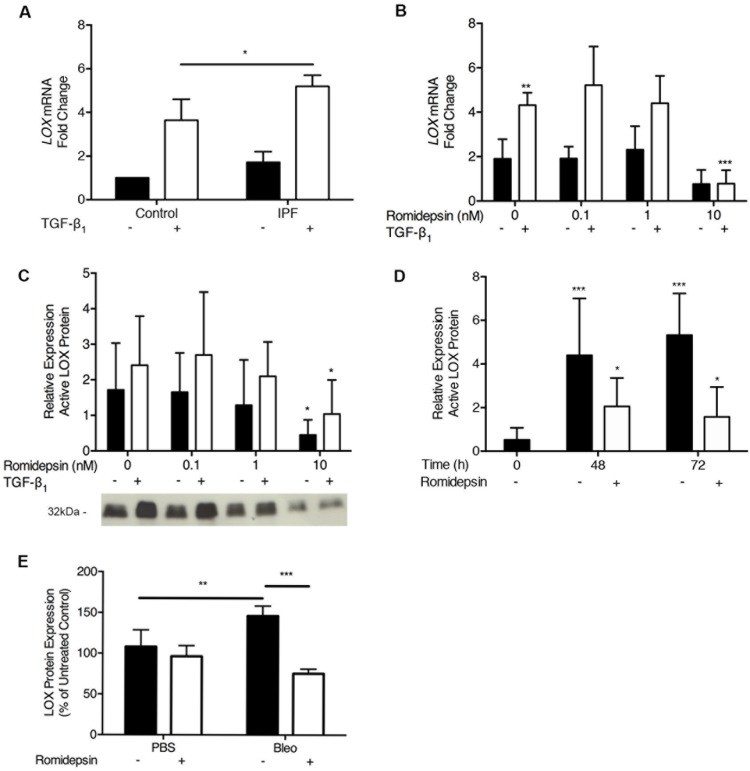
Inhibition of LOX expression by Romidepsin: IPF or normal fibroblasts were cultured in DMEM/FBS in the absence (solid bars) or presence (open bars) of TGF-β1 and ± romidepsin for 48h Cells were lysed for RNA analysis by RT-qPCR and cell-conditioned media harvested for western blot analysis. **A**. LOX mRNA expression in the absence or presence of TGF-β1 after 48h. Data are presented as mean + SD (*n* = 3 per group; one-way ANOVA) **B**. LOX mRNA expression in IPF fibroblasts with increasing concentrations of romidepsin in the presence or absence of TGF-β1. Data were normalized to the lowest expressing fibroblast cell line from a control donor at baseline. **C**. & **D**. Western blot analysis of active LOX protein (32 kDa) secreted from IPF fibroblasts, demonstrating dose and time responses, respectively, to romidepsin. Representative blot is shown in panel C; data were normalized to total protein loaded using a Ponceau stain and expressed relative to a positive control to control for between blot variation. Data in **B**.-**D**. are presented as mean + SD (*n* = 4 IPF donors, each performed in duplicate). **E**. Semi quantitative analysis of LOX protein measured in homogenized mouse lung as prepared as previously described in Figure 7. Data are presented as mean + SD (*n* = 4 per group within one experiment; two-way ANOVA with Tukey's multiple comparisons). *P < 0.05, **P < 0.01, ***P < 0.001. Where there are no lines to indicate the statistical comparison, it is between the starred bar and its equivalent baseline non-romidepsin treated control.

LOX is a secreted enzyme that initiates crosslinking of collagens and elastin and is regulated by TGF-β_1_ [24, 25]. We detected significantly higher levels of LOX mRNA in IPF fibroblasts treated with TGF-β_1_ compared to control fibroblasts (Figure 8A). This effect of TGF-β_1_ was dose-dependently reduced by romidepsin (Figure 8B). As the primary location of LOX is extracellular [41], we also evaluated changes at the protein level in fibroblast cell-conditioned media. Pro- and active forms of LOX were both detectable, with the 32kDa active form being predominant under the conditions studied. At 48h, 10nM romidepsin significantly suppressed active LOX protein expression in IPF fibroblasts, both in the absence or presence of TGF-β_1_ (Figure 8C). Romidepsin had a long-lasting effect, preventing the accumulation of LOX in conditioned media from cells treated with TGF-β_1_, up to 72h after a single drug treatment (Figure 8D). Consistent with these *in vitro* findings, Western blotting of lung homogenates from bleomycin treated mice revealed that LOX protein was suppressed by romidepsin (Figure 8E), suggesting that suppression of LOX protein levels may be a biomarker of romidepsin activity *in vivo*.

**Figure 9:**
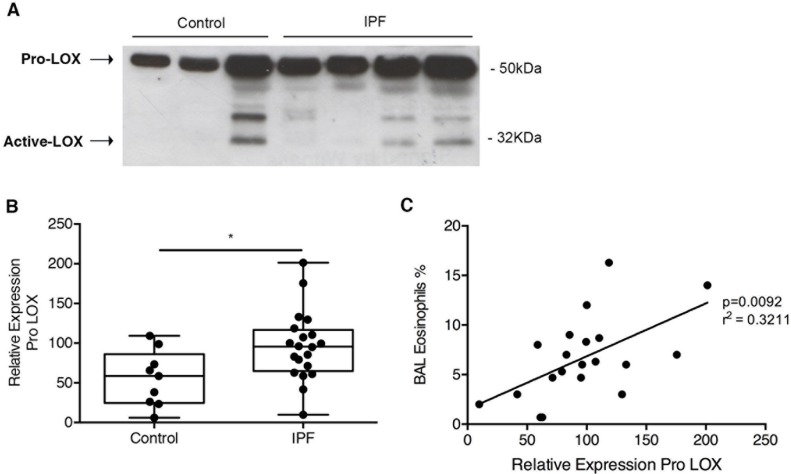
LOX was increased in IPF and correlated with increased eosinophils: BALF from IPF patients and control donors were analyzed by western blot for LOX expression **A**. Representative blot with **B**. semi-quantitative analysis of Pro LOX expression normalized to Ponceau stain for total protein, and expressed relative to a positive control. **C**. Regression analysis showing the correlation between BAL eosinophils and relative levels of Pro-LOX. Data are presented as mean + SD (*n* = 9 control and 20 IPF donors). **P < 0.01.

To assess whether LOX was increased in IPF lungs, BALF was obtained from 9 normal subjects and 20 subjects with a diagnosis of IPF established by a multi-disciplinary team according to ATS/ERS/JRS/ALAT criteria [6]. Western blotting revealed a major protein band at 50kDa, consistent with the presence of pro-LOX (Figure 9A). This band was detected in all samples and semi-quantitative analysis revealed significant up-regulation in BALF from IPF patients compared to controls (Figure 9B). Although the age of the control donors was lower than the IPF subjects, there was no significant correlation between age and pro-LOX expression in the IPF group (R^2^ = 0.07 and *P* = 0.27). Interestingly, BALF eosinophil count correlated closely with pro-LOX densitometry levels (Figure 9C). In addition to the presence of pro-LOX, other smaller bands between 32-50kDa were detected in some samples. These bands most likely represent glycosylated forms of active LOX, or partially processed forms of the enzyme [41]. Active LOX was minimal in control BALF with only 1 of 9 (11%) samples exhibiting strong immunoreactivity; in contrast, active LOX was present in 12 of 20 (60%) IPF BALF samples. Those subjects with active LOX also had higher pro-LOX densitometry.

## DISCUSSION

IPF is a fatal disease characterized by uncontrolled fibroblast proliferation, myofibroblast differentiation and excessive ECM deposition suggesting that multiple signaling pathways may be involved in disease pathogenesis. Since epigenetic mechanisms have been implicated in IPF [10–12] and we have shown that the HDAC inhibitor SpA has powerful anti-fibrotic effects [17], here we report the potential of romidepsin as a candidate therapy for IPF. This anti-cancer agent holds great promise and could be readily progressed into an IPF clinical trial, as it is a licensed and established therapy in CTCL.

Romidepsin exhibited potent anti-proliferative and anti-fibrotic properties when tested on human IPF fibroblasts *in vitro* without any significant harmful effect on ATII cells. Romidepsin also showed anti-fibrotic activity in a murine model of pulmonary fibrosis and its effects were associated with increased histone acetylation consistent with the drug acting as an epigenetic modulator. Finally, to further support development of romidepsin as a novel therapeutic approach for IPF, we have identified LOX as a potential candidate biomarker for a proof-of-mechanism clinical trial of romidepsin.

The 2011 ATS/ERS/JRS/ALAT statement on IPF [6] highlighted the failure of numerous ‘single pathway’ agents to show efficacy in clinical trials of IPF. HDACs increase acetylation of histones and non-histone proteins, including transcription factors, to regulate gene expression. Thus, by targeting multiple genes and pathways, HDAC inhibitors offer a novel therapeutic strategy to address the unmet need in IPF. Consistent with this, our data demonstrate that romidepsin potently inhibits multiple fibrogenic processes including fibroblast proliferation, myofibroblast differentiation and ECM synthesis. Increased expression of p21^waf1^ leading to G1/S cell cycle arrest explains the effect of romidepsin on cell proliferation, while suppression of *HDAC4* expression would account for its effect on myofibroblast differentiation [14]. Using 2D and 3D culture models, as well as an *in vivo* model of pulmonary fibrosis, romidepsin suppressed interstitial collagen production as well as expression of LOX, whose activity is required for collagen cross-linking. Thus, romidepsin may suppress the secretion of collagen and its cross-linking thereby reducing the stiffness of the ECM that has been observed in IPF lung [42]. This should promote matrix turnover to facilitate resolution of aberrant wound healing responses.

The anti-proliferative effects of romidepsin occurred at low nanomolar concentrations, significantly lower than those required with other HDAC inhibitors. In our *in vitro* studies, the effects of romidepsin appeared to be cytostatic, in line with our previous work on SpA [17] and similar work in cancer cells [43]. In contrast with its potent effects on fibroblasts, romidepsin had little effect on ATII cell number or lamellar bodies, suggesting minimal toxicity on this cell typ*e*. Lamellar bodies are storage organelles of pulmonary surfactant and their degeneration has been reported as a marker of toxicity and injury for the lung epithelium [44–46]. Our findings are consistent with studies of romidepsin in CTCL where no pulmonary toxicity was reported [20]. Furthermore, in phase II trials in lung cancer, romidepsin shifted the global gene expression profile of the cancer cells towards that of normal epithelial cells [22].

Romidepsin completed phase I trials prior to fast-track FDA-approval for use in CTCL in 2004, and its dose-limiting side effects include thrombocytopenia, nausea and fatigue [47]. Following from our findings *in vitro*, it may be possible that lower doses of romidepsin will be required in an IPF application compared to CTCL as cytostatic, rather than cytotoxic, effects are desired. Furthermore, in the phase II trial of lung cancer, the maximum steady-state plasma DP concentrations ranged from 384 to 1,114 ng/mL [22]. As these doses are more than 2 orders of magnitude greater than those required to inhibit growth of IPF fibroblasts and ECM biosynthesis by myofibroblasts, it seems likely that lower doses of romidepsin may provide an anti-fibrotic effect with much lower associated toxicity.

In taking forward a novel IPF therapeutic into clinical development, one of the significant challenges is the lack of sensitive clinical end-points resulting in large trials of long duration [48]. One way to improve confidence in a potential therapeutic is demonstration of target engagement (proof of mechanism) in the lung by linking preclinical data to clinical readouts. We have shown that we can detect elevated levels of pro-LOX, in the BALF of IPF patients and that active LOX is significantly elevated in patients with a diagnosis of IPF. Since romidepsin can reduce LOX expression, our studies suggest that a reduction in levels of LOX proteins in IPF BALF could provide early evidence of proof-of-mechanism for romidepsin even before effects on lung function or patient survival are evident. We also found an interesting correlation between LOX and the number of eosinophils detected in the BALF of IPF patients. Previous studies have implicated eosinophils in lung fibrosis due to their effect on fibroblast proliferation and differentiation *via* TGF-β_1_ [49, 50]. Therefore, it might also be of interest to monitor eosinophil numbers following romidepsin treatment.

In summary, the potent anti-proliferative and anti-fibrotic properties of romidepsin *in vitro* and *in vivo* strongly support progression of this FDA-approved HDAC inhibitor towards a clinical trial to evaluate its potential as a new therapy for IPF. Identification of LOX as secreted protein whose levels are increased in IPF lung and whose production is modulated by romidepsin support its evaluation as a companion biomarker with the actual physiological end-points for assessing the early proof-of-mechanism of romidepsin in study patients.

## MATERIALS AND METHODS

### Compounds

Romidepsin (FK228) was from Selleck Chemicals (Munich, Germany) or Celgene Corporation, Summit, NJ, USA (ISTODAX^®^, Romidepsin for injection). Solid was dissolved in dimethylsulphoxide (DMSO) (Sigma-Aldrich, Gillingham, UK) and then further diluted into aqueous solution for use. A vehicle control (DMSO or saline, as appropriate) was run in each experiment.

### Primary fibroblast culture

Parenchymal lung fibroblast cultures were established from explant tissue of patients with IPF obtained by video-assisted thoracoscopic lung biopsy at University Hospital Southampton (*n* = 3) or from macroscopically normal resected lung tissue (*n* = 3) following ethical approval (LREC: 07/H0607/73 and LREC: 08/H0502/32) and informed written consent. This work has been carried out in accordance with the Declaration of Helsinki. Fibroblasts were cultured in Dulbecco's Modified Eagle's Medium (DMEM) supplemented with 10% foetal bovine serum (FBS), 50 units/ml penicillin, 50μg/ml streptomycin, 2mM L-glutamine, 1mM sodium pyruvate and 1x non-essential amino acids (DMEM/FBS) (all from Life Technologies, Paisley, UK). 3D cultures were performed as previously described [17].

### Primary alveolar type II (ATII) cell culture

ATII cells were isolated from macroscopically normal regions of surgically resected lung tissue obtained with ethical approval (LREC: 08/H0502/32) and informed written consent. This work has been carried out in accordance with the Declaration of Helsinki. The parenchymal lung tissue was processed according to the method of Witherden et al. [51]. Briefly, the lung tissue was perfused with 0.9% saline solution and instilled with 0.25% Trypsin (Sigma Aldrich, Poole, UK) at 37°C for 45 min. After trypsin digestion, the tissues were finely sliced in the presence of newborn calf serum (NCS) and DNase (250mg/ml), and then cells were filtered by sequential passage through a 400μm metal mesh and 40μm nylon filter. The cells were re-suspended in DCCM-1 medium (Biological Industries Ltd, Kibbutz Beit-Haemek, Israel) supplemented with 1% penicillin, 1% streptomycin and 1% L-glutamine, and incubated at 37°C in a humidified incubator for 2h in tissue culture flasks to allow differential adherence and removal of contaminating mononuclear cells. The alveolar epithelial cells were re-suspended in fresh DCCM-1 supplemented with 10% NCS, 1% penicillin, 1%streptomycin and 1% L-glutamine and plated on collagen 1 (PureCol 5005-b, Advanced BioMatrix Inc., California USA) coated 96 well plates at 60% density; after 72h purity was tested by staining for alkaline phosphatase.

### Methylene blue proliferation assay

Fibroblasts (1×10^3^ cells/well in DMEM/FBS) were treated in the absence or presence of 5ng/ml TGF-β_1_ (PeproTech, London, UK) for 24h prior to treatment with romidepsin. TGF-β_1_ 5ng/ml had previously been determined (unpublished data) as the optimum concentration to induce myofibroblast differentiation, a key event in fibrosis. Cells were fixed in formal saline at 48, 72 and 144h before staining with methylene blue [52]. Cell number, determined by direct cell counting, was proportional to absorbance at 630nm after stained cells were eluted in 100μl 1:1 0.1% HCl and ethanol.

### Cell proliferation by direct cell counting

Human ATII cells (*n* = 3, 1 male and 2 female) and IPF fibroblasts (*n* = 3, 3 male) were plated at 60-70% confluence and then treated and then treated with romidepsin. After 72h, the cells were fixed in 90% ethanol and nuclei stained with 4′,6-diamidino-2-phenylindole (DAPI). Cell nuclei were imaged by tile scan fluorescent microscopy of the entire well and counted using ImageJ software.

### Reverse transcription and quantitative PCR (RTqPCR)

Fibroblasts (1×10^5^/well in DMEM/FBS) were pre-treated with medium alone, or with 5 or 10ng/ml TGF-β_1_ for 24h prior to treatment with Romidepsin. At 48h, RNA was isolated using Trizol (Life Technologies, Paisley, UK) and any genomic DNA contamination removed by DNase treatment (Ambion, Warrington, UK) before cDNA synthesis. Changes in mRNA expression using the following primers were analyzed by qPCR and normalized to the housekeeping genes ubiquitin C and phospholipase A2 *(UBC* and *A2)* using the ΔΔCT method [53]: *CDKN1A* (NM_000389), Fwd 5′-TCCAGCCGACCTTCCTCATC-3′ and Rev 5′-GCCTCTACTGCCACCATCT-3′; ACTA2 (NM_001613), Fwd 5′-AAGCACAGAGCAAAAGAGGAAT-3′ and Rev 5′-ATGTCGTCCCAGTTGGTGAT-3′; *HDAC4* (NM_006037), Fwd 5′-GCCACCATTCACCTCTGTAAT-3′ and Rev 5′-AAATCCACCCACACAAAACAAG’-3; *COL1A1* (NM_000088), Fwd 5′-AGACAGTGATTGAATACAAAACCA-3′ and Rev 5′-GGAGTTTACAGGAAGCAGACA-3′; *COL3A1* (NM_000090), Fwd 5′-GTCCCGCTGGCATTCCTG-3′ and Rev 5′-CTCTCCTTTGGCACCATTCTTAC-3′ *LOX* (NM_002317), Fwd 5′-GATATAGTCTAAATTAGCAAAGCACATAG-3′ and Rev 5′-ATTACGCAGCACAGTCCTTG-3′ . For murine fibrosis studies, left lungs were collected into RNA later®. For RNA extraction, the lungs were transferred into Trizol reagent homogenized using a Ribolyser (Savant FastPrep FP12, Qbiogene Cedex, France) and then RNA extracted as above. Changes in mRNA expression were analyzed using the following primers: *Fn1* (NM_010233), Fwd 5′-AAGAGGACGTTGCAGAGCTA-3′ and Rev 5′- AGACACTGGAGACACTGACTAA; *Col3a1* (NM_009929), Fwd 5′-CCGTTGAGTCCGTCTTTGC-3′ and Rev 5′-ATATGCCCACAGCCTTCTAC-3′; *Col1a1* (NM_007742)*,* Fwd 5′-GTCCACGTAGTTGGCGACTTC-3′ and Rev 5′-CCGTTGAGTCCGTCTTTGC-3′; *Lox* (NM_010728) Fwd 5′-GACATTCGCTACACAGGACAT-3′ and Rev 5′-AACACCAGGTACGGCTTTATC-3′. Data were normalized to the housekeeping gene *Gapdh* using the ΔΔCT method [53]. All PCR reagents (including reverse transcription kits) were purchased from PrimerDesign Ltd (Southampton, UK).

### Soluble collagen expression (sircol assay)

Briefly, fibroblasts were pelleted at 1×10^6^/ml and placed in 2ml DMEM/FBS in the absence or presence of 5ng/ml TGF-β_1_ and without or with 5nM romidepsin. Supernatants were exchanged every second day and harvested conditioned media clarified by centrifugation for analysis of soluble collagen. Soluble collagen was measured in cell-free culture supernatants using the sircol assay according to the manufacturer's instructions (Sircol Collagen Assay Kit; Biocolor Ltd, Carrickfergus, UK). Briefly, culture medium was treated with an isolation and concentration reagent overnight on an ice-water mix at 4°C. Samples were centrifuged at 12,000rpm for 10 minutes at 4°C and the supernatant removed. Sirius red dye was added to the pellet to form a complex with the collagen. Unbound dye was removed and the pellet solubilized in alkali reagent for measurement of absorbance at 555nm to allow quantification by a reference standard curve.

### Western blot analyses

Fibroblasts were cultured as for RNA analysis. After incubation, cells were lysed into SDS sample buffer for SDS-PAGE and cell conditioned media clarified by centrifugation before concentrating secreted proteins using StrataClean resin at 1:10 (Agilent Technologies, Wokingham, UK) [54]. For murine fibrosis studies, right lungs were snap frozen and then homogenized in normal saline containing protease inhibitors (Roche, Burgess Hill, UK) before solubilization in SDS sample buffer for SDS-PAGE.

Western blotting of cellular or lung lysates was performed for acetyl histone H3, 1:20000 (Merck Millipore, Watford, UK), and of cell-conditioned medium for secreted pro-LOX and LOX, (1:20, Sigma-Aldrich, Poole, UK) using enhanced chemiluminescence detection (ECL+, GE Healthcare, Buckinghamshire, UK). In the case of acetyl H3, membranes were stripped and reprobed using pan histone H3 antibodies (Merck Millipore, Watford, UK); for LOX quantitation, image analysis of Ponceau Red stain (Sigma-Aldrich, Poole, UK) was used a loading control. Densitometry was used for semi-quantitative analysis of the data.

### Flow cytometry analysis

Cell cycle was quantitatively determined by flow cytometry analysis. Fibroblasts (1×10^3^cells/well in DMEM/FBS) were cultured for 48 hours and then treated with romidepsin. Cells were collected after 48h treatment and fixed in cold (−20°C) 70% ethanol, treated with RNase A 42μg/ml, and stained with 7μg/ml of PI. A FACS Calibur (Becton Dickinson) was used to quantify the percentage of cells in each cell cycle phase by acquiring 10,000 events per sample. Analysis of cell cycle distribution was performed with BD CellQuest software.

### CARS microscopy

ATII cells were plated at 60% density on collagen I coated multi-well chamber slides (Grace Bio-Labs, Stratech Scientific Limited, Newmarket, UK) for high-resolution imaging. After attachment, the cells were treated with 5nM romidepsin and the cells were cultured for 72h before fixation in 4% paraformaldehyde (PFA). A purpose-built CARS microscope comprising a Chameleon (Coherent) and Compact OPO (APE Berlin) coupled to an inverted Nikon Ti-U 2000 microscope (Nikon, Kingston upon Thames, UK) was used to acquire images. Images were acquired with a 40x (NA: 1.2) water-immersion objective (Nikon). Symmetric CH_2_ vibrations at the Raman vibrational frequency of 2845 cm^−1^ were used to selectively image surfactant lipid droplets (LDs) in the cells [55]. Images (*n* = 7-11) were acquired on random cell areas for each condition. LDs were quantified using a code written in MATLAB (MathWorks, Cambridge, UK). The number of pixels with intensities exceeding the threshold were counted and were compared with the overall cell area in pixels to obtain a ratio of LDs per cell area.

### Transmission electron microscopy

Human primary alveolar type II cells were plated on porous filter inserts (pore size 0.4μm; Corning 3470, Fisher Scientific UK Ltd, Loughborough, UK) coated with collagen I and treated with romidepsin 5nM. After 72h, cells were fixed using 3% glutaraldehyde and 4% formaldehyde in 0.1M PIPES buffer pH 7.2, post fixed with 1% osmium tetroxide in 0.1M PIPES buffer pH 7.2 and then with 2% aqueous uranyl acetate. The samples were dehydrated in graded ethanols and embedded in SPURR resin. Ultrathin sections were obtained using an ultra-microtome (Reichert OMu3 Ultra, Type 700141, Leica, Milton Keynes, UK) and grids were stained with Reynolds lead reagent. Micrograph pictures were acquired using transmission electron microscopy (Hitachi H7000, Maidenhead, UK).

### Collection and processing of bronchoalveolar lavage fluid (BALF)

BALF was collected by fiberoptic bronchoscopy performed according to current guidelines [56]. BALF from IPF patients was collected at the Royal Brompton Hospital, London (*n* = 20, LREC:10/H0720/12) and healthy volunteers at the NIHR Respiratory Biomedical Research Unit, Southampton University Hospitals NHS Trust (*n* = 9, LREC:09/H0502/91). All patients met the ATS/ERS/JRS/ALAT criteria for the diagnosis of IPF [6]; subject characteristics are given in Table 1. Samples of BALF containing 20μg protein were processed using Strataclean resin and the eluted protein analyzed for LOX protein by western blotting, as described above.

**Table 1:** Patient characteristics of control donors and IPF patients used in BALF analysis

Controls	Age	Sex	Smoking Hx	% FEV_1_	% FVC	% T_LCO_
Donor 1	51	F	Y	83.8	103.5	83.0
Donor 2	47	M	N	108.2	110.7	83.0
Donor 3	65	M	N	91.4	97.3	87.0
Donor 4	37	M	N	110.5	97.5	90.0
Donor 5	62	M	Y	97.1	115.6	109.0
Donor 6	51	F	Y	92.2	106.1	70.0
Donor 7	53	M	Y	128.1	144.7	75.0
Donor 8	46	N/A	N	N/A	N/A	N/A
Donor 9	39	M	N	90.9	94.7	76.1
**Mean**	50.1	6/2/1a	5/4b	100.3	108.8	84.14
**SE**	3.1			5.1	5.7	4.2

IPF patients	Age	Sex	Smoking Hx	% FEV_1_	% FVC	% T_LCO_
IPF 1	54	M	Y	79.0	73.1	69.0
IPF 2	59	M	N	67.4	79.0	38.2
IPF 3	76	F	N	86.3	92.8	53.0
IPF 4	65	M	N	72.2	65.4	38.7
IPF 5	64	M	Y	75.0	74.7	39.4
IPF 6	60	M	N/A	74.7	71.4	28.5
IPF 7	72	F	N	123.6	120.5	57.1
IPF 8	73	M	N	64.0	56.0	40.0
IPF 9	46	M	N	63.0	59.0	30.0
IPF 10	49	M	Ex	57.6	52.7	42.9
IPF 11	58	M	Ex	54.5	49.3	37.0
IPF 12	70	M	N	72.1	65.9	50.5
IPF 13	70	M	N	83.4	84.2	39.3
IPF 14	74	M	N/A	80.8	77.1	32.4
IPF 15	70	M	Y	76.9	74.9	36.4
IPF 16	71	M	N	82.2	85.9	54.4
IPF 17	74	M	Ex	78.7	79.7	32.9
IPF 18	66	M	Ex	65.1	62.0	56.1
IPF 19	61	M	Ex	73.9	68.8	30.6
IPF 20	54	M	Y	70.6	82.0	73.5
**Mean**	64.3	18/2a	9/4/5b	75.1	73.7	44
**SE**	2			3.2	3.6	2.9

^a^Male/Female/Not available, bNever/Yes/Ex smoker. Abbreviaions: Smoking Hx = smoking history, N/A = not available, % FEV1 = forced expiratory volume in one second expressed as % predicted, %FVC = forced vital capacity expressed as % predicted, %TLCO = transfer factor of the lung for carbon monoxide expressed as % predicted, SE = standard error.

### Bleomycin mouse model

All animal procedures were performed in compliance with the US Department of Health and Human Services Guide for the Care and Use of Laboratory Animals. The study was approved and supervised by the University of Rochester University Committee on Animal Resources. Adult male C57BL/6J mice (The Jackson Laboratory, Bar Harbor, ME) were treated, at 8 weeks of age, with 2U/kg pharmaceutical grade bleomycin (Hospira, Lake Forest, IL) in 40μl PBS by oropharyngeal aspiration [57], or PBS alone (controls). Mice were group housed in shoebox cages (3-5 mice per cage) on a 12 hours light/dark cycle and received food and water ad libitum; mice were randomly assigned to treatment groups. The dose of romidepsin (2mg/kg IP in saline) used for the study was based on previous cancer studies in mice [58]. To assess early gene expression changes associated with fibrosis, mice were injected with a single dose of romidepsin (2mg/kg IP in saline) or saline alone (control) on day 3 after bleomycin and harvested on day 7. The left lung was snap frozen for later Western blot analysis and the right lung was stored in RNAlater® (Qiagen, Valencia, CA) for later determination of mRNA levels. To assess the effect of romidepsin on fibrosis, mice were treated with bleomycin as described, and treated with romidepsin (2mg/kg IP) on days 3, 7, 11, and 15; and harvested on day 21. The right lung was dissected away from the left lung and other tissue, the wet weight was determined, and the lung was then used for hydroxyproline assay as described [57]. The left lung was inflated and fixed with neutral buffered formalin. Sections (5μm) were stained with Gomori's Trichrome, and whole slide images were taken with an Olympus WS110 whole slide imager.

### Statistical analysis

Differences between two means were assessed using either Students T test or Mann-Whitney U test for parametric or non-parametric data respectively. For multiple comparisons, analysis of variance (ANOVA) was employed with a Tukey or Dunnett's post-test. *p* < 0.05 were accepted as statistically significant. *p < 0.05, **p < 0.01, *** *p* < 0.001.

## Abbreviations

ACTA2: Actin Alpha 2
α-SMA: alpha-smooth muscle actin
ATII: alveolar type II cells
BALF: Bronchoalveolar lavage fluid
CARS: coherent anti-Stokes Raman scattering spectroscopy
CDKN1A: Cyclin-Dependent Kinase Inhibitor 1A
COL3A1: collagen 3 A1
ECM: extracellular matrix
Esbriet^®^: pirfenidone
FDA: Food and Drug Administration Agency
HDAC: Histone deacetylase
HDAC3: histone deacetylase 3
HDAC4: histone deacetylase 4
IPF: idiopathic pulmonary fibrosis
LOX: lysyl oxidase
Ofev^®^: nintedanib
TGF-β: transforming growth factor beta

## References

[1] Selman M, Pardo A (2014). Revealing the pathogenic and aging-related mechanisms of the enigmatic idiopathic pulmonary fibrosis. an integral model. Am J Respir Crit Care Med.

[2] Raghu G, Weycker D, Edelsberg J, Bradford WZ, Oster G (2006). Incidence and prevalence of idiopathic pulmonary fibrosis. Am J Respir Crit Care Med.

[3] Noble PW, Albera C, Bradford WZ, Costabel U, Glassberg MK, Kardatzke D, King TE, Lancaster L, Sahn SA, Szwarcberg J, Valeyre D, du Bois RM, Group CS, CAPACITY Study Group (2011). Pirfenidone in patients with idiopathic pulmonary fibrosis (CAPACITY): two randomised trials. Lancet.

[4] King TE, Bradford WZ, Castro-Bernardini S, Fagan EA, Glaspole I, Glassberg MK, Gorina E, Hopkins PM, Kardatzke D, Lancaster L, Lederer DJ, Nathan SD, Pereira CA (2014). A phase 3 trial of pirfenidone in patients with idiopathic pulmonary fibrosis. N Engl J Med.

[5] Richeldi L, du Bois RM, Raghu G, Azuma A, Brown KK, Costabel U, Cottin V, Flaherty KR, Hansell DM, Inoue Y, Kim DS, Kolb M, Nicholson AG (2014). Efficacy and safety of nintedanib in idiopathic pulmonary fibrosis. N Engl J Med.

[6] Raghu G, Collard HR, Egan JJ, Martinez FJ, Behr J, Brown KK, Colby TV, Cordier JF, Flaherty KR, Lasky JA, Lynch DA, Ryu JH, Swigris JJ (2011). An official ATS/ERS/JRS/ALAT statement: idiopathic pulmonary fibrosis: evidence-based guidelines for diagnosis and management. Am J Respir Crit Care Med.

[7] Sime PJ, O'Reilly KM (2001). Fibrosis of the lung and other tissues: new concepts in pathogenesis and treatment. Clin Immunol.

[8] Hinz B (2010). The myofibroblast: paradigm for a mechanically active cell. J Biomech.

[9] Vancheri C, Failla M, Crimi N, Raghu G (2010). Idiopathic pulmonary fibrosis: a disease with similarities and links to cancer biology. Eur Respir J.

[10] Coward WR, Watts K, Feghali-Bostwick CA, Knox A, Pang L (2009). Defective histone acetylation is responsible for the diminished expression of cyclooxygenase 2 in idiopathic pulmonary fibrosis. Mol Cell Biol.

[11] Huang SK, Scruggs AM, Donaghy J, Horowitz JC, Zaslona Z, Przybranowski S, White ES, Peters-Golden M (2013). Histone modifications are responsible for decreased Fas expression and apoptosis resistance in fibrotic lung fibroblasts. Cell Death Dis.

[12] Korfei M, Skwarna S, Henneke I, MacKenzie B, Klymenko O, Saito S, Ruppert C, von der Beck D, Mahavadi P, Klepetko W, Bellusci S, Crestani B, Pullamsetti SS (2015). Aberrant expression and activity of histone deacetylases in sporadic idiopathic pulmonary fibrosis. Thorax.

[13] Lakshmaiah KC, Jacob LA, Aparna S, Lokanatha D, Saldanha SC (2014). Epigenetic therapy of cancer with histone deacetylase inhibitors. J Cancer Res Ther.

[14] Guo W, Shan B, Klingsberg RC, Qin X, Lasky JA (2009). Abrogation of TGF-beta1-induced fibroblast-myofibroblast differentiation by histone deacetylase inhibition. Am J Physiol Lung Cell Mol Physiol.

[15] Wang Z, Chen C, Finger SN, Kwajah S, Jung M, Schwarz H, Swanson N, Lareu FF, Raghunath M (2009). Suberoylanilide hydroxamic acid: a potential epigenetic therapeutic agent for lung fibrosis?. Eur Respir J.

[16] Sanders YY, Hagood JS, Liu H, Zhang W, Ambalavanan N, Thannickal VJ (2014). Histone deacetylase inhibition promotes fibroblast apoptosis and ameliorates pulmonary fibrosis in mice. Eur Respir J.

[17] Davies ER, Haitchi HM, Thatcher TH, Sime PJ, Kottmann RM, Ganesan A, Packham G, O'Reilly KM, Davies DE (2012). Spiruchostatin A inhibits proliferation and differentiation of fibroblasts from patients with pulmonary fibrosis. Am J Respir Cell Mol Biol.

[18] Ueda H, Nakajima H, Hori Y, Fujita T, Nishimura M, Goto T, Okuhara M (1994). FR901228, a novel antitumor bicyclic depsipeptide produced by Chromobacterium violaceum No. 968. I. Taxonomy, fermentation, isolation, physico-chemical and biological properties, and antitumor activity. J Antibiot (Tokyo).

[19] Greshock TJ, Johns DM, Noguchi Y, Williams RM (2008). Improved total synthesis of the potent HDAC inhibitor FK228 (FR-901228). Org Lett.

[20] Mottet D, Castronovo V (2008). Histone deacetylases: target enzymes for cancer therapy. Clin Exp Metastasis.

[21] Barbarotta L, Hurley K (2015). Romidepsin for the Treatment of Peripheral T-Cell Lymphoma. J Adv Pract Oncol.

[22] Schrump DS, Fischette MR, Nguyen DM, Zhao M, Li X, Kunst TF, Hancox A, Hong JA, Chen GA, Kruchin E, Wright JJ, Rosing DR, Sparreboom A (2008). Clinical and molecular responses in lung cancer patients receiving Romidepsin. Clin Cancer Res.

[23] Otterson GA, Hodgson L, Pang H, Vokes EE, Cancer Leukemia Group B (2010). Phase II study of the histone deacetylase inhibitor Romidepsin in relapsed small cell lung cancer (Cancer and Leukemia Group B 30304). J Thorac Oncol.

[24] Robins SP (2007). Biochemistry and functional significance of collagen cross-linking. Biochem Soc Trans.

[25] Shanley CJ, Gharaee-Kermani M, Sarkar R, Welling TH, Kriegel A, Ford JW, Stanley JC, Phan SH (1997). Transforming growth factor-beta 1 increases lysyl oxidase enzyme activity and mRNA in rat aortic smooth muscle cells. J Vasc Surg.

[26] Counts DF, Evans JN, Dipetrillo TA, Sterling KM, Kelley J (1981). Collagen lysyl oxidase activity in the lung increases during bleomycin-induced lung fibrosis. J Pharmacol Exp Ther.

[27] Sakamoto M, Murawaki Y, Hirayama C (1987). Serum lysyl oxidase activity in patients with various liver diseases. Gastroenterol Jpn.

[28] Di Donato A, Ghiggeri GM, Di Duca M, Jivotenko E, Acinni R, Campolo J, Ginevri F, Gusmano R (1997). Lysyl oxidase expression and collagen cross-linking during chronic adriamycin nephropathy. Nephron.

[29] Sivakumar P, Gupta S, Sarkar S, Sen S (2008). Upregulation of lysyl oxidase and MMPs during cardiac remodeling in human dilated cardiomyopathy. Mol Cell Biochem.

[30] Calderwood CJ, Davies ER, Jones MG, Hoile L, Maher TM, O'Reilly KM, Davies DE (2012). The histone deacetylase inhibitor FK228 has potent anti‐fibrotic properties in idiopathic pulmonary fibrosis.

[31] Davies ER, Calderwood CJ, Jones MG, Hoile L, Thatcher TH, Sime PJ, Maher TM, O'Reilly KM, Davies DE (2013). The Depsipeptide HDAC Inhibitor FK228 Has Potent Anti-Fibrotic Properties In IPF Fibroblasts And Decreases The Expression Of Lysyl Oxidase A Potential IPF Biomarker. American Journal of Respiratory and Critical Care Medicine.

[32] Davies ER, Ganesan A, Packham GK, Thatcher TH, Sime PJ, O'Reilly KM, Davies DE (2011). The Depsipeptide HDAC Inhibitor FK228 (Romidepsin) has Anti-Fibrotic Properties in Fibrotic Primary Pulmonary Fibroblasts. American Journal of Respiratory and Critical Care Medicine.

[33] Davies ER, Haitchi HM, Thatcher TH, Sime PJ, Kottmann RM, Ganesan A, Packham G, O'Reilly KM, Davies DE (2012). Spiruchostatin A Inhibits Proliferation And Differentiation Of Primary Fibroblasts From Patients With Interstitial Lung Disease. ATS International Conference. Am J Respir Cell Mol Biol.

[34] Conforti F, Davies ER, Jones MG, Alzetani A, Skipp P, Warner J, Molyneaux P, Smart D, Tetley TD, Havelock T, Maher TM, Thatcher TH, Mahajan S (2016). Evaluation of romidepsin (FK228) as a potential therapy for idiopathic pulmonary fibrosis (IPF). European Respiratory Society.

[35] Johnstone RW (2002). Histone-deacetylase inhibitors: novel drugs for the treatment of cancer. Nat Rev Drug Discov.

[36] Crabb SJ, Howell M, Rogers H, Ishfaq M, Yurek-George A, Carey K, Pickering BM, East P, Mitter R, Maeda S, Johnson PW, Townsend P, Shin-ya K (2008). Characterisation of the in vitro activity of the depsipeptide histone deacetylase inhibitor spiruchostatin A. Biochem Pharmacol.

[37] Evans MJ, Cabral LJ, Stephens RJ, Freeman G (1975). Transformation of alveolar type 2 cells to type 1 cells following exposure to NO2. Exp Mol Pathol.

[38] Wang HW, Fu Y, Huff TB, Le TT, Wang H, Cheng JX (2009). Chasing lipids in health and diseases by coherent anti-Stokes Raman scattering microscopy. Vib Spectrosc.

[39] Walters DM, Kleeberger SR (2008). Mouse models of bleomycin-induced pulmonary fibrosis. Curr Protoc Pharmacol.

[40] Moore BB, Hogaboam CM (2008). Murine models of pulmonary fibrosis. Am J Physiol Lung Cell Mol Physiol.

[41] Trackman PC, Bedell-Hogan D, Tang J, Kagan HM (1992). Post-translational glycosylation and proteolytic processing of a lysyl oxidase precursor. J Biol Chem.

[42] Booth AJ, Hadley R, Cornett AM, Dreffs AA, Matthes SA, Tsui JL, Weiss K, Horowitz JC, Fiore VF, Barker TH, Moore BB, Martinez FJ, Niklason LE, White ES (2012). Acellular normal and fibrotic human lung matrices as a culture system for in vitro investigation. Am J Respir Crit Care Med.

[43] Bolden JE, Peart MJ, Johnstone RW (2006). Anticancer activities of histone deacetylase inhibitors. Nat Rev Drug Discov.

[44] Schmitz G, Müller G (1991). Structure and function of lamellar bodies, lipid-protein complexes involved in storage and secretion of cellular lipids. J Lipid Res.

[45] Nakatani Y, Nakamura N, Sano J, Inayama Y, Kawano N, Yamanaka S, Miyagi Y, Nagashima Y, Ohbayashi C, Mizushima M, Manabe T, Kuroda M, Yokoi T, Matsubara O (2000). Interstitial pneumonia in Hermansky-Pudlak syndrome: significance of florid foamy swelling/degeneration (giant lamellar body degeneration) of type-2 pneumocytes. Virchows Arch.

[46] Magnani KL, Cataneo DC, Capelozzi VL, Defaveri J, Hasimoto EN, Cataneo AJ (2012). Lung morphology and growth of rats exposed to tobacco smoke and alcohol. Acta Cir Bras.

[47] Mizutani H, Hiraku Y, Tada-Oikawa S, Murata M, Ikemura K, Iwamoto T, Kagawa Y, Okuda M, Kawanishi S (2010). Romidepsin (FK228), a potent histone deacetylase inhibitor, induces apoptosis through the generation of hydrogen peroxide. Cancer Sci.

[48] Nathan SD, Meyer KC (2014). IPF clinical trial design and endpoints. Curr Opin Pulm Med.

[49] Levi-Schaffer F, Garbuzenko E, Rubin A, Reich R, Pickholz D, Gillery P, Emonard H, Nagler A, Maquart FA (1999). Human eosinophils regulate human lung- and skin-derived fibroblast properties in vitro: a role for transforming growth factor beta (TGF-beta). Proc Natl Acad Sci USA.

[50] Huaux F, Liu T, McGarry B, Ullenbruch M, Xing Z, Phan SH (2003). Eosinophils and T lymphocytes possess distinct roles in bleomycin-induced lung injury and fibrosis. J Immunol.

[51] Witherden IR, Vanden Bon EJ, Goldstraw P, Ratcliffe C, Pastorino U, Tetley TD (2004). Primary human alveolar type II epithelial cell chemokine release: effects of cigarette smoke and neutrophil elastase. Am J Respir Cell Mol Biol.

[52] Oliver MH, Harrison NK, Bishop JE, Cole PJ, Laurent GJ (1989). A rapid and convenient assay for counting cells cultured in microwell plates: application for assessment of growth factors. J Cell Sci.

[53] Livak KJ, Schmittgen TD (2001). Analysis of relative gene expression data using real-time quantitative PCR and the 2(−Delta Delta C(T)) Method. Methods.

[54] Ziegler J, Vogt T, Miersch O, Strack D (1997). Concentration of dilute protein solutions prior to sodium dodecyl sulfate-polyacrylamide gel electrophoresis. Anal Biochem.

[55] McKenzie Z, Kendall M, Mackay RM, Whitwell H, Elgy C, Ding P, Mahajan S, Morgan C, Griffiths M, Clark H, Madsen J (2015). Surfactant protein A (SP-A) inhibits agglomeration and macrophage uptake of toxic amine modified nanoparticles. Nanotoxicology.

[56] Meyer KC, Raghu G, Baughman RP, Brown KK, Costabel U, du Bois RM, Drent M, Haslam PL, Kim DS, Nagai S, Rottoli P, Saltini C, Selman M (2012). An official American Thoracic Society clinical practice guideline: the clinical utility of bronchoalveolar lavage cellular analysis in interstitial lung disease. Am J Respir Crit Care Med.

[57] Lakatos HF, Burgess HA, Thatcher TH, Redonnet MR, Hernady E, Williams JP, Sime PJ (2006). Oropharyngeal aspiration of a silica suspension produces a superior model of silicosis in the mouse when compared to intratracheal instillation. Exp Lung Res.

[58] Sasakawa Y, Naoe Y, Inoue T, Sasakawa T, Matsuo M, Manda T, Mutoh S (2003). Effects of FK228, a novel histone deacetylase inhibitor, on tumor growth and expression of p21 and c-myc genes in vivo. Cancer Lett.

